# Case Report: Multidisciplinary management with dental autotransplantation for rehabilitation of a post-traumatic edentulous maxillary hemi-segment in a growing patient: transverse maxillary development after 10 years

**DOI:** 10.3389/fdmed.2026.1825107

**Published:** 2026-05-04

**Authors:** Eduardo Anitua, Laura Piñas, Mohammad H. Alkhraisat

**Affiliations:** 1 University Institute for Regenerative Medicine and Oral Implantology (UIRMI), Vitoria, Spain; 2 BTI Biotechnology Institute, Vitoria, Spain

**Keywords:** autotransplantation, tooth autotransplantation, growing patient, maxillary growth, dental trauma

## Abstract

**Introduction:**

Dental autotransplantation has re-emerged as a valuable biological option for tooth replacement in growing patients, mainly due to its ability to preserve the periodontal ligament and maintain alveolar bone. Nevertheless, its potential influence on transverse maxillary development over the long term remains insufficiently explored. The aim of this report was to present a 10-year follow-up of a multidisciplinary rehabilitation, with particular attention to transverse maxillary growth.

**Case report:**

A growing patient who suffered severe dentoalveolar trauma, with loss of all teeth in the upper right quadrant, was treated through a staged multidisciplinary approach. Two unerupted mandibular premolars were autotransplanted to the maxilla, followed by orthodontic alignment and, once growth was completed, implant-supported rehabilitation. Throughout the follow-up period, the transplanted teeth remained functional and vital. One implant developed marginal bone loss after seven years and was successfully replaced after regenerative therapy. At 10 years, both implants and autotransplanted teeth were clinically stable, with CBCT confirming preservation of the regenerated alveolar ridge.

**Results:**

Transverse measurements of the maxilla showed similar dimensions between the treated and contralateral sides at both canine and molar levels. These findings suggest that transverse development of the affected hemi-maxilla was not compromised.

**Conclusion:**

Within the limitations of a single case, this report supports the concept that dental autotransplantation may play a role beyond tooth replacement, potentially contributing to the maintenance of transverse maxillary growth during development. This reinforces its value as a biologically driven strategy in complex cases involving growing patients.

## Introduction

Dental autotransplantation, also referred to as autologous transplantation, is defined as the surgical transfer of a tooth from one site to another within the same individual. This may involve the relocation of an ectopic, impacted, or erupted tooth into a fresh extraction socket or a surgically prepared recipient site (1–10). As a biological and cost-effective therapeutic option, autotransplantation has been proposed for the replacement of teeth with a poor prognosis, particularly in young patients, in whom it offers unique long-term advantages related to growth, tissue preservation, and function (1–10).

The concept of tooth transplantation is not new and has been described in dental practice for several centuries. Historical records indicate that tooth transplantation was already performed in the late 18th and early 19th centuries, particularly in London, where allotransplantation between individuals was relatively common, although associated with limited longevity, with an average survival of approximately six years (11–13). The modern era of dental autotransplantation, however, emerged in Scandinavia during the 1950s and 1960s (11), when systematic clinical and experimental research began to refine surgical protocols and biological principles. A major milestone was reached in 1974 with the publication by Slagsvold and Bjercke (14) of a detailed protocol for the transplantation of premolars with incomplete root development. This work was subsequently reinforced by the extensive longitudinal studies conducted by Andreasen and colleagues, with follow-up periods extending up to 38 years, which laid the foundations for standardized techniques, prognostic assessment, and long-term monitoring (15). Although early clinical reports described relatively modest success rates, often around 50%, subsequent advances in surgical technique, case selection, and biological understanding have progressively consolidated autotransplantation as a predictable treatment modality (16, 17). Contemporary studies report survival rates ranging from 68% to 100%, values that are comparable to those reported for dental implants, even in follow-ups extending up to 41 years (6, 17–22). In particular, single-stage autotransplantation of teeth with incomplete root development has been shown to be highly predictable, with reported survival rates exceeding 97% at two years (22, 23). Findings from meta-analysis further support the high performance of tooth autotransplantation, indicating an overall survival rate of 97.9%, with 1-year 2-year, 5-year and 10-year survival rates of 98%, 97%, 95.9% and 96.9%, respectively (22, 24–27). Notably, teeth with open apices consistently demonstrate survival rates exceeding 95% at both 5 and 10 years (27–29). Conversely, biological complications such as replacement resorption or ankylosis may still occur, highlighting the importance of long-term clinical and radiographic follow-up (5, 14, 15, 30–36).

Prognosis of autotransplanted tooth is strongly influenced by several factors, including the stage of root development, atraumatic handling of the donor tooth and periodontal ligament, characteristics of the recipient site, and patient-related variables (20, 25, 37–39). Teeth transplanted at an immature stage—typically with two-thirds to three-quarters of root development—demonstrate the most favourable outcomes, with a reported relative risk of failure of 0.3 compared with teeth with a closed apex (24, 40). The occurrence of root reportion has been higher in premolars with fully developed roots than premolars with three-quarters of root development (41). Teeth with closed apices usually require adjunctive endodontic treatment to reduce the risk of inflammatory root resorption (20, 42). Conversely, the availability of insufficient alveolar bone to support the autotransplanted tooth would be an obstacle to the success of tooth autotransplantation (43). Furthermore, poor oral hygiene and lack of self-motivation are contraindications for tooth autotransplantation (15, 41, 44, 45).

Thus, the biological basis for the success of dental autotransplantation lies in the preservation and regeneration of periodontal ligament and pulpal tissues (46, 47). This regenerative potential allows for the maintenance of alveolar bone volume and gingival architecture, supports normal dentoalveolar growth in young patients, and permits subsequent orthodontic tooth movement (8, 9, 35, 48). Moreover, in growing patients, autotransplantation may function as a biological space maintainer, preserving hard and soft tissues and maintaining future restorative options, thereby reinforcing its clinical relevance in contemporary dentistry (18, 21, 49).

Recent technological developments have further enhanced the precision and predictability of autotransplantation procedures. Advances in cone-beam computed tomography and rapid 3D prototyping have enabled the fabrication of accurate surgical templates for recipient site preparation immediately prior to transplantation (43, 50–52). This approach has significantly reduced extraoral time of the donor tooth, a critical factor for periodontal ligament cell survival, and is expected to further improve clinical outcomes by avoiding excessive compression of the tooth apex (2, 6, 51–55).

In the present report, we provide an update of a case originally published in 2017 (43) involving a young patient who sustained severe dentoalveolar trauma with loss of all teeth in the upper right quadrant. The case was managed using a comprehensive multidisciplinary approach in which dental autotransplantation played a central role, complemented by orthodontic, restorative, aesthetic, and implant-based treatments. This update extends the follow-up to 10 years, allowing assessment of long-term clinical and radiographic outcomes.

Beyond this extended follow-up, the case also offers the opportunity to explore transverse maxillary development in a hemi-maxilla rehabilitated through dental autotransplantation during active craniofacial growth. Most published reports have focused mainly on survival and functional outcomes, whereas information on transverse skeletal development is still limited (56–60). In this context, the present case provides additional clinical insight into the potential of autotransplanted teeth to contribute to alveolar modelling and maxillary growth over time, which is particularly relevant in growing patients.

## Case report

The present clinical case represents the continuation of the follow-up of a previously published report in which outcomes were documented up to four years (43). As a brief summary of the initial phase, the patient was a 13-year-old male at the start of treatment who had suffered severe trauma following a bicycle accident, resulting in the loss of all teeth in the first quadrant, with the exception of tooth 17, which had not yet erupted. As part of the initial treatment, autotransplantation of two unerupted mandibular premolars (teeth 35 and 45) was performed into the positions corresponding to teeth 11 and 14 (Figure 1a). Once stability of the transplanted teeth had been confirmed, root development had been completed, and eruption of tooth 17 had occurred, orthodontic treatment was initiated to align the arch, perform esthetic camouflage of tooth 11 (the autotransplanted premolar), and rehabilitate the remaining edentulous areas through the placement of dental implants. At the completion of the initial four-year follow-up, complete stabilization of the maxillary arch had been achieved. At that time, the only pending event was the eruption of tooth 18 into the position of tooth 17, which had migrated during orthodontic treatment to occupy the position corresponding to tooth 16.

**Figure 1:**
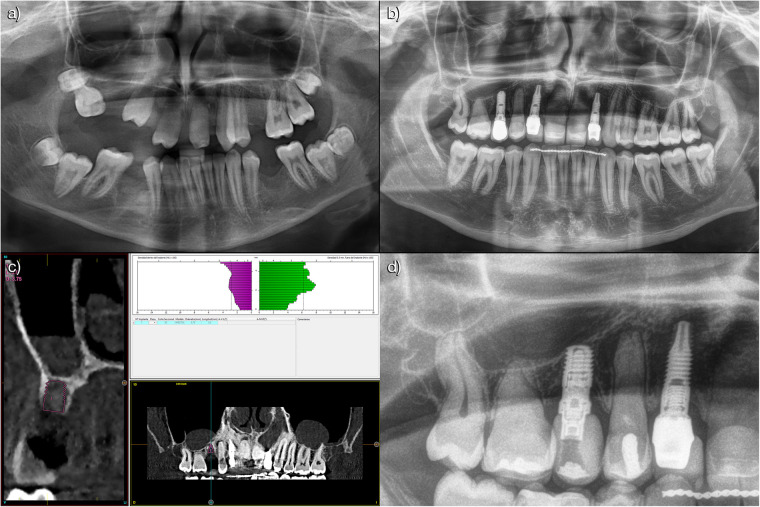
**(a)** final radiograph after dental autotransplantation showing the two mandibular premolars positioned in the locations they will occupy in the first quadrant, corresponding to the areas of teeth 11 and 14. **(b)** At 19 years of age (seven years of follow-up), clinical and radiographic examination revealed advanced marginal bone loss around the implant placed in the premolar region. **(c)** Planning of the new implant, to be placed in the socket regenerated with PRGF-Endoret. Implant dimensions were 3.75 mm in diameter and 5.5 mm in length. **(d)** Six months later, the implant was loaded using a unitary transepithelial abutment (Unit®, Biotechnology Institute, Vitoria, Spain) and a lithium disilicate (E-max) crown cemented to an interface.

Following the four-year follow-up previously reported, the patient continued under regular clinical and radiographic surveillance throughout late adolescence. During this period, the implant-supported restorations and autotransplanted teeth remained functional and asymptomatic, allowing assessment of their behavior during the final stages of craniofacial growth.

At 19 years of age (seven years of follow-up), clinical and radiographic examination revealed advanced marginal bone loss around the implant placed in the premolar region (Figure 1b). Given the unfavorable prognosis, atraumatic explantation was performed. The defect was managed using regenerative procedures with plasma rich in growth factors, enabling preservation of the remaining bone architecture.

After six weeks, sufficient bone regeneration was achieved to allow placement of a new implant measuring 3.75 mm in diameter and 5.5 mm in length (Figure 1c), thereby avoiding the need for adjunctive procedures such as sinus elevation. Six months later, the implant was loaded using a unitary transepithelial abutment (Unit®, Biotechnology Institute, Vitoria, Spain) and a lithium disilicate (E-max) crown cemented to an interface (Figure 1d).

Subsequent follow-up demonstrated favorable evolution, and at 20 years of age, (eight years of follow-up) definitive ceramic veneers were placed on the central incisors to accommodate changes associated with residual craniofacial growth (Figures 2a,b). After completion of the definitive prosthetic phase, the patient was followed for an additional two years. At the 10-year follow-up, all implants and autotransplanted teeth were clinically asymptomatic and functional. Sectional CBCT images confirmed long-term maintenance of peri-implant and alveolar bone levels, as well as stability of the regenerated maxillary ridge (Figures 3a–c).

**Figure 2:**
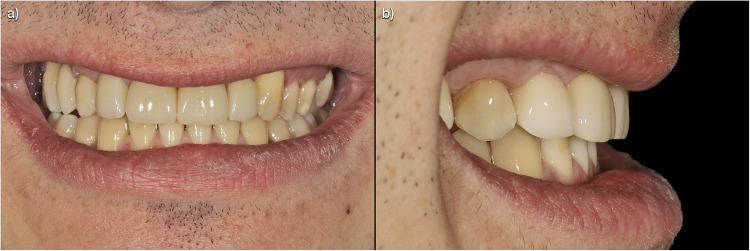
Final appearance of the patient with new ceramic veneers after completion of craniofacial growth (20 years of age). **(a)** front view, **(b)** lateral view.

**Figure 3:**
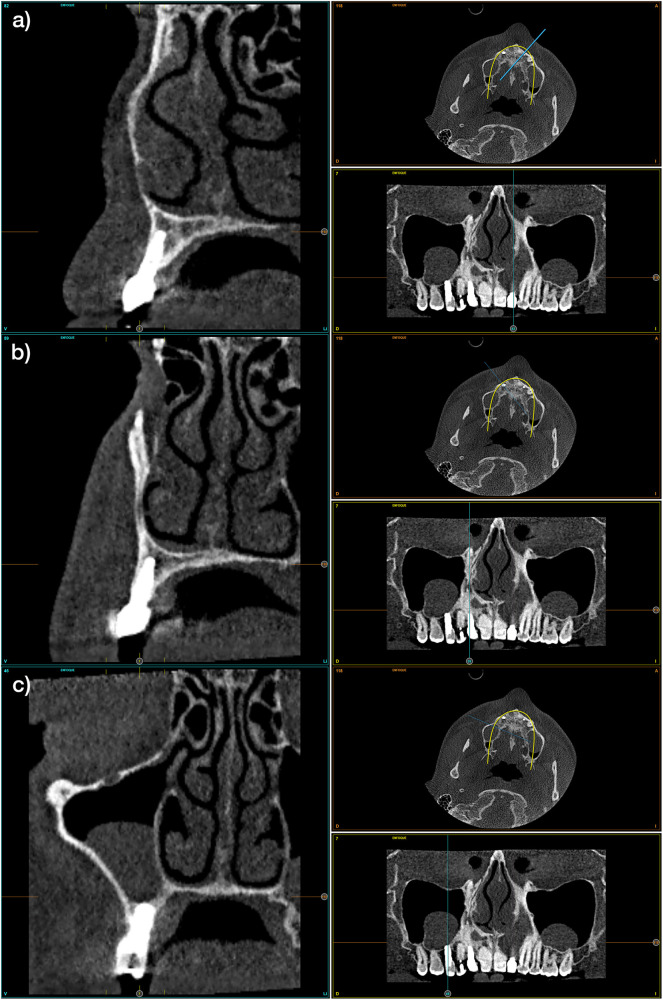
**(a–c)** control cone-beam computed tomography images showing stability of the implants placed at the end of the patient's follow-up period.

**Figure 4:**
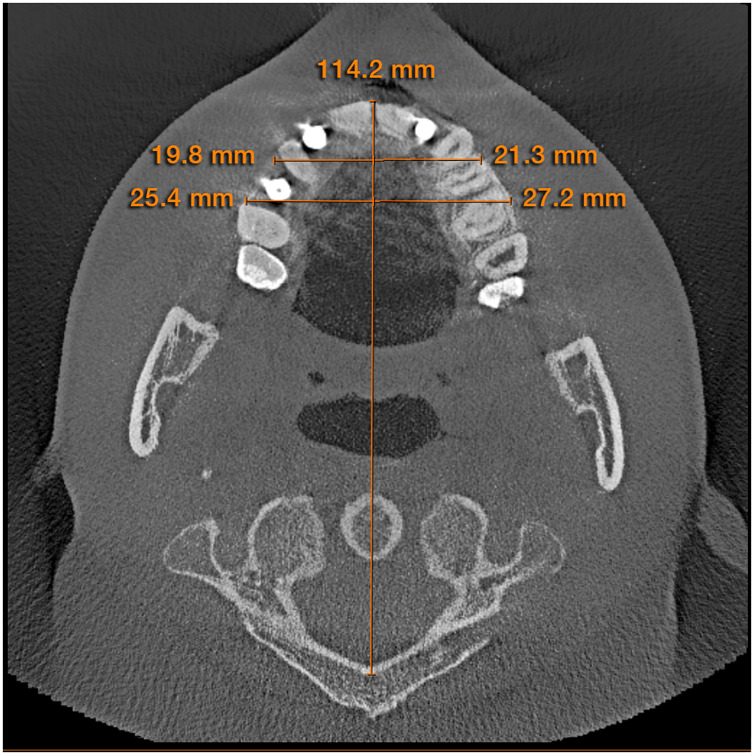
Coronal section showing the transverse development of the maxilla in the area rehabilitated by dental autotransplantation compared with the contralateral side, where growth occurred without intervention. Both hemi-maxillae exhibit a nearly equivalent transverse width.

At the 10-year follow-up, transverse maxillary development was assessed using cone-beam computed tomography (CBCT). Measurements were performed on standardized coronal sections, defining hemi-maxillary width as the distance from the midpalatal raphe to the outer cortical boundary of the alveolar process, using a perpendicular reference line. Two anatomical levels were evaluated: the canine region and the first molar region.

The treated quadrant, corresponding to the side rehabilitated with dental autotransplantation, showed transverse dimensions comparable to those of the contralateral side. Specifically, the maxillary width at the canine level measured 19.8 mm on the autotransplanted side compared with 21.3 mm on the contralateral side, while at the first molar level the width was 25.4 mm on the treated side and 27.2 mm on the contralateral side.

Taken together, these findings indicate similar transverse dimensions between both sides, suggesting that normal hemi-maxillary development was maintained in the region rehabilitated with autotransplantation.

From a biological perspective, this outcome supports the concept that the presence of functional teeth, preserved periodontal ligament, and physiological occlusal loading may contribute to maintaining and stimulating normal alveolar and maxillary growth during development (15, 19, 30, 61, 62). In growing patients, dental autotransplantation has been shown to preserve alveolar bone volume and to allow adaptive modelling of the alveolar process in harmony with craniofacial growth, in contrast to dental implants, which lack a periodontal ligament and do not provide the same biological stimulus for transverse or vertical bone development (15, 56, 62, 63). Previous clinical and radiographic studies have demonstrated that autotransplanted teeth can support continued alveolar bone growth and remodelling, thereby acting as biological placeholders that guide skeletal development during growth (15, 30, 45). In the present case, it is reasonable to assume that, in the absence of transplanted teeth and subsequent implant-supported rehabilitation, the affected hemi-maxilla would have shown reduced transverse development, as has been reported in edentulous areas not exposed to functional stimulation.

Written informed consent was obtained from the patient's legal guardian (parent) for the publication of any potentially identifiable images or data included in this article during the period of minority. Upon reaching the age of 18 years, written informed consent was subsequently obtained from the patient.

## Discussion

Dental autotransplantation is a well-established technique for the replacement of missing teeth, particularly in young patients and in the anterior region (43). The international literature describes autotransplantation procedures in both anterior and posterior regions of the maxilla and mandible, although these approaches clearly serve different purposes and indications (45, 64, 65).

In the posterior region, autotransplantation is most used to replace failed molars due to caries, fractures, or unsuccessful restorative treatments, typically using another erupting molar as the donor tooth, with third molars being the most frequently selected option (65). In these procedures, survival rates of the transplanted teeth are generally high but variable. Systematic reviews report that, in contemporary clinical series, one-year survival rates are usually very high (96%–98%), whereas five-year survival rates show a broader range (81%–98%), reflecting heterogeneity in root maturity, surgical technique, and criteria used to define “success” (e.g., absence of resorption or ankylosis vs. mere tooth retention) (66). In the specific subgroup of immature third molars transplanted to the molar region, a recent meta-analysis estimated a pooled survival rate of approximately 91.9% (95% CI: 85.1–96.7) and a success rate of approximately 90.2% (95% CI: 79.8–97.1), supporting the notion that, with appropriate case selection and control of key biological variables, such as periodontal ligament handling, extraoral time, and flexible fixation, the procedure is clinically predictable (67). In contrast, when molars with closed apices (fully developed roots) are analysed, survival rates tend to be lower and the risk of biological complications, such as inflammatory or replacement resorption (ankylosis), becomes more clinically relevant, as reflected in studies focusing on mature third molars and prognostic factor analyses (68, 69).

In the anterior aesthetic region, autotransplantation is most frequently performed using premolars as donor teeth, particularly in young patients, as they allow biological and functional rehabilitation of the anterior segment and subsequent orthodontic and restorative integration when required (63). Compared with multirooted teeth, premolars, typically single-rooted, tend to allow more favorable surgical manipulation and a technically less complex procedure, which has been associated with predictable clinical outcomes in series and reviews focused on transplantation to the anterior region (14, 15, 31, 32, 34, 70–72). In this context, several studies have identified the stage of root development of the donor tooth as a key prognostic factor for long-term outcomes (73). The literature shows that teeth with incomplete root formation, typically between one-half and three-quarters of root development, have a higher probability of clinical success and a lower incidence of biological complications than teeth with fully developed roots (22, 26, 34, 73). This has been correlated with a greater capacity for pulpal revascularization and periodontal regeneration, favoring continued root development and long-term functional stability of the transplanted tooth (33, 74). Consistently, clinical series and systematic reviews have reported that immature teeth tend to show higher survival and success rates and a reduced need for endodontic treatment compared with mature teeth, reflecting a more favourable outcome when transplantation is performed during the root development phase (6, 15, 24, 26, 29, 38, 66, 75).

In the present case, the transplanted premolars, with more than two-thirds of root development and open apices at the time of transplantation, showed a favourable clinical evolution throughout the follow-up period. During the 10-year observation period, no signs of ankylosis were detected, and both teeth responded adequately to orthodontic treatment, suggesting preservation of functional periodontal ligament activity. Likewise, no pulpal complications were observed, as neither premolar required endodontic treatment and both maintained pulpal vitality throughout the entire clinical and radiographic follow-up. Additionally, placement of the transplanted premolars contributed to a favourable transverse development of the maxilla, comparable to that expected under normal functional conditions.

In line with these observations, one of the most relevant findings of the present case is the comparable transverse development between the treated and untreated hemi-maxillae after 10 years. This observation supports the concept that autotransplanted teeth, through preservation of the periodontal ligament and exposure to functional loading, may contribute to maintaining physiological bone modelling during growth. Recent CBCT-based studies have also reported preservation of alveolar bone volume and continued remodelling around autotransplanted teeth, supporting their role as biologically active structures rather than passive replacements (56). In contrast, dental implants lack a periodontal ligament and therefore do not adapt to craniofacial growth, which may even lead to disturbances in bone development if placed prematurely (76).

Long-term clinical studies further indicate that autotransplanted teeth can maintain functional periodontal ligament activity and respond to orthodontic forces, both of which are essential for proper transverse and vertical development of the maxilla (15, 30, 59). However, despite these well-established biological advantages, quantitative data specifically addressing transverse maxillary development remain limited. In this context, the present case provides additional clinical insight suggesting that autotransplantation may help preserve maxillary development in post-traumatic defects.

Within this context, the present case provides additional clinical insight suggesting that autotransplantation may help preserve transverse maxillary growth in post-traumatic defects, reinforcing its potential role not only as a replacement strategy but also as a biologically driven approach capable of supporting craniofacial development in growing patients.

In growing patients, the presence of a functional tooth not only restores occlusion but also contributes to the preservation and adaptation of the alveolar ridge during development (34, 56, 63, 77, 78). This biological advantage has been described in clinical series and narrative reviews highlighting how autotransplantation of immature teeth supports continued alveolar bone development in harmony with craniofacial growth, in contrast to dental implants, whose placement is generally postponed until near completion of growth to avoid occlusal and bone remodelling complications (63, 77, 78). Since implants lack a periodontal ligament and physiological adaptive capacity, their premature placement in growing patients may limit transverse and vertical alveolar bone development, whereas transplanted teeth can integrate biologically into active growth processes (62, 76, 79). In the final phase of growth, implant placement allowed completion of the patient's functional rehabilitation while preserving the alveolar ridge previously developed through biological and orthodontic treatments (61, 79). In this context, implants played a key role as a definitive solution once craniofacial growth had stabilized, avoiding the complications described when implants are placed at earlier stages. Complementarily, orthodontic treatment was essential for balancing both arches and achieving a functional and stable long-term occlusion (2, 31, 48, 80). Overall, these findings highlight the importance of a multidisciplinary approach in complex post-traumatic cases, where coordination between specialties is essential to achieve predictable and biologically favourable outcomes (3, 58).

## Conclusions

The present case demonstrates that dental autotransplantation represents a an effective therapeutic option in young patients with complex tooth loss, particularly when performed during the active growth phase and with appropriate donor tooth selection. The use of premolars with incomplete root development allowed not only functional and aesthetic replacement of the affected area but also preservation and stimulation of alveolar bone development, creating a favourable environment for subsequent definitive rehabilitation (Figure 4). The sequential combination of autotransplantation, orthodontic treatment, and delayed implant placement after stabilization of craniofacial growth resulted in stable functional, aesthetic, and biological outcomes over the long term. These findings highlight the importance of individualized treatment planning and a multidisciplinary approach in the management of severe dentoalveolar trauma in growing patients, aiming to maximize biological potential and optimize long-term clinical results.

## Data Availability

The raw data supporting the conclusions of this article will be made available by the authors, without undue reservation.

## References

[1] Tair JAA Rahhal A. Tooth autotransplantation in orthodontic patients. J Contemp Dent Pract. 2017. 11(3):63–70. 10.5005/jcdp-11-3-63 20461326

[2] Abella Sans F Ribas F Doria G Roig M Durán-Sindreu F. Guided tooth autotransplantation in edentulous areas post-orthodontic treatment. J Esthet Restor Dent. (2021) 33:685–91. 10.1111/jerd.12786 34002459

[3] Ferraresso LdO Besegato JF Maler GL Takahashi R Stábile GAV Hoeppner MG. Tooth autotransplantation and resin composite reshaping as a multidisciplinary approach for treating dental agenesia: a 10-year follow-up. J Esthet Restor Dent. (2025) 37:307–13. 10.1111/jerd.13284 39054618

[4] Ashurko I Vlasova I Yaremchuk P Bystrova O. Autotransplantation of teeth as an alternative to dental implantation. BMJ Case Rep. (2020) 13:e234889. 10.1136/bcr-2020-234889 32532914 PMC7295376

[5] Doomen R Barendregt D Temmerman L De Roo N De Pauw G. Premolar autotransplantation to the anterior maxilla region—aesthetic outcome and patient satisfaction. Dent Traumatol. (2025) 41:534–47. 10.1111/edt.13057 40071571

[6] Tan BL Tong HJ Narashimhan S Banihani A Nazzal H Duggal MS. Tooth autotransplantation: an umbrella review. Dent Traumatol. (2023) 39:2–29. 10.1111/edt.12836 36898857

[7] Fernandes S Al Habibullah M Nalam GS Nair PP. Think before you extract—a case of tooth autotransplantation. BMJ Case Rep. (2011) 2011:bcr0620114380. 10.1136/bcr.06.2011.4380 22679047 PMC3176353

[8] Ong DCV Goh P Dance G. Anterior tooth autotransplantation: a case series. Aust Dent J. (2023) 68:202–15. 10.1111/adj.12966 37415408

[9] Ong D Itskovich Y Dance G. Autotransplantation: a viable treatment option for adolescent patients with significantly compromised teeth. Aust Dent J. (2016) 61:396–407. 10.1111/adj.12420 27029674

[10] Stange KM Lindsten R Bjerklin K. Autotransplantation of premolars to the maxillary incisor region: a long-term follow-up of 12–22 years. Eur J Orthod. (2016) 38:508–15. 10.1093/ejo/cjv078 26490395

[11] Cross D El-Angbawi A McLaughlin P Keightley A Brocklebank L Whitters J Developments in autotransplantation of teeth. Surgeon. (2013) 11:49–55. 10.1016/j.surge.2012.10.003 23142342

[12] Dokova AF Lee JY Mason M Moretti A Reside G Christensen J. Advancements in tooth autotransplantation. J Am Dent Assoc. (2024) 155:475–83. 10.1016/j.adaj.2024.01.006 38530692

[13] Kim MS Lee HS Nam OH Choi SC. Autotransplantation: a reliable treatment modality for severely malpositioned teeth. J Clin Pediatr Dent. (2017) 41:388–91. 10.17796/1053-4628-41.5.388 28872984

[14] Slagsvold O Bjercke B. Autotransplantation of premolars with partly formed roots. A radiographic study of root growth. Am J Orthod. (1974) 66:355–66. 10.1016/0002-9416(74)90046-3 4529559

[15] Andreasen JO Paulsen HU Yu Z Bayer T Schwartz O. A long-term study of 370 autotransplanted premolars. Part II. Tooth survival and pulp healing subsequent to transplantation. Eur J Orthod. (1990) 12:14–24. 10.1093/EJO/12.1.14 2318259

[16] Kuo HY Lo YJ Huang YW. New era of tooth autotransplantation. Part I: thoughts on clinical aspects. J Dent Sci. (2024) 19:8–10. 10.1016/J.JDS.2023.10.027 38303853 PMC10829753

[17] Kakde K Rajanikanth K. Tooth autotransplantation as an alternative biological treatment: a literature review. Cureus. (2022) 14:e30491. 10.7759/cureus.30491 36420247 PMC9678114

[18] Tsukiboshi M Yamauchi N Tsukiboshi Y. Long-term outcomes of autotransplantation of teeth: a case series. J Endod. (2019) 45:S72–83. 10.1016/j.joen.2019.05.016 31623912

[19] Tsukiboshi M. Autotransplantation of teeth: requirements for predictable success. Dent Traumatol. (2002) 18:157–80. 10.1034/j.1600-9657.2002.00118.x 12442825

[20] Cremona M Bister D Sherriff M Abela S. Prognostic factors, outcomes, and complications for dental autotransplantation: an umbrella review. Eur J Orthod. (2024) 46(1):cjad067. 10.1093/ejo/cjad067 37995718

[21] Akhlef Y Schwartz O Andreasen JO Jensen SS. Autotransplantation of teeth to the anterior maxilla: a systematic review of survival and success, aesthetic presentation and patient-reported outcome. Dent Traumatol. (2018) 34:20–7. 10.1111/edt.12379 28990720

[22] Sicilia-Pasos J Kewalramani N Peña-Cardelles JF Salgado-Peralvo AO Madrigal-Martínez-Pereda C López-Carpintero Á. Autotransplantation of teeth with incomplete root formation: systematic review and meta-analysis. Clin Oral Investig. (2022) 26:3795–805. 10.1007/s00784-022-04435-8 35258700

[23] Verweij JP Wes JT van Teeseling RA Becking AG. Pre-autotransplantation alveolar process augmentation and premolar autotransplantation as a treatment method for single tooth replacement in adolescents. Int J Oral Maxillofac Surg. (2021) 50:1632–7. 10.1016/j.ijom.2021.04.010 33985865

[24] Almpani K Papageorgiou SN Papadopoulos MA. Autotransplantation of teeth in humans: a systematic review and meta-analysis. Clin Oral Investig. (2015) 19:1157–79. 10.1007/s00784-015-1473-9 25903060

[25] Machado LA do Nascimento RR Ferreira DMTP Mattos CT Vilella OV. Long-term prognosis of tooth autotransplantation: a systematic review and meta-analysis. Int J Oral Maxillofac Surg. (2016) 45:610–7. 10.1016/j.ijom.2015.11.010 26696138

[26] Rohof ECM Kerdijk W Jansma J Livas C Ren Y. Autotransplantation of teeth with incomplete root formation: a systematic review and meta-analysis. Clin Oral Investig. (2018) 22:1613–24. 10.1007/s00784-018-2408-z 29525924 PMC5906482

[27] Atala-Acevedo C Abarca J Martínez-Zapata MJ Díaz J Olate S Zaror C. Success rate of autotransplantation of teeth with an open apex: systematic review and meta-analysis. J Oral Maxillofac Surg. (2017) 75:35–50. 10.1016/j.joms.2016.09.010 27725103

[28] Lazic EK Jakovljevic A Widbiller M. Autotransplantation of teeth with open apices may result in high survival rates but evidence is limited. J Evid Based Dent Pract. (2024) 24:101984. 10.1016/j.jebdp.2024.101984 38821655

[29] Juslin J Jääsaari P Teerijoki-Oksa T Suominen A Thorén H. Survival of autotransplanted teeth with open apices: a retrospective cohort study. J Oral Maxillofac Surg. (2020) 78:902.e1–e9. 10.1016/j.joms.2020.02.018 32184086

[30] Czochrowska EM Stenvik A Bjercke B Zachrisson BU. Outcome of tooth transplantation: survival and success rates 17–41 years posttreatment. Am J Orthod Dentofacial Orthop. (2002) 121:110–9. 10.1067/mod.2002.119979 11840123

[31] Tanaka T Deguchi T Kageyama T Kanomi R Inoue M Foong KWC. Autotransplantation of 28 premolar donor teeth in 24 orthodontic patients. Angle Orthodontist. (2008) 78:12–9. 10.2319/120706-495.1 18193971

[32] Czochrowska EM Stenvik A Album B Zachrisson BU. Autotransplantation of premolars to replace maxillary incisors: a comparison with natural incisors. Am J Orthod Dentofacial Orthop. (2000) 118:592–600. 10.1067/mod.2000.110521 11113791

[33] Züger M Mukkadam K Filippi A Schmidlin PR. Periodontal status after autologous tooth transplantation (ATT) of premolars and wisdom teeth. Swiss Dent J. (2024) 134:54–71. 10.61872/sdj-2024-04-05 39501737

[34] Miyai T Cho J Tai K Park JH. Autotransplanted premolars with incomplete root formation in a growing patient with multiple missing teeth. Am J Orthod Dentofacial Orthop. (2020) 158:587–98. 10.1016/j.ajodo.2019.05.024 32839074

[35] Velozo C Nogueira LR Nogueira Filho LR Capistrano A de Albuquerque DS. Tooth autotransplantation using an interdisciplinary approach to rehabilitation in a young patient: case report with 7-year follow-up. Dent Traumatol. (2021) 37:521–30. 10.1111/edt.12637 33269534

[36] Yoshino K Ishizuka Y Sugihara N Kariya N Namura D Noji I Gender difference in tooth autotransplantation with complete root formation: a retrospective survey. J Oral Rehabil. (2013) 40:368–74. 10.1111/joor.12038 23438017

[37] Aoyama S Yoshizawa M Niimi K Sugai T Kitamura N Saito C. Prognostic factors for autotransplantation of teeth with complete root formation. Oral Surg Oral Med Oral Pathol Oral Radiol. (2012) 114(5):S216–28. 10.1016/j.oooo.2011.09.037 22705233

[38] Yang S Jung BY Pang NS. Outcomes of autotransplanted teeth and prognostic factors: a 10-year retrospective study. Clin Oral Investig. (2019) 23:87–98. 10.1007/s00784-018-2412-3 29525925

[39] Jang Y Choi YJ Lee SJ Roh BD Park SH Kim E. Prognostic factors for clinical outcomes in autotransplantation of teeth with complete root formation: survival analysis for up to 12 years. J Endod. (2016) 42:198–205. 10.1016/j.joen.2015.10.021 26686824

[40] Kim E Jung JY Cha IH Kum KY Lee SJ. Evaluation of the prognosis and causes of failure in 182 cases of autogenous tooth transplantation. Oral Surg Oral Med Oral Pathol Oral Radiol Endodontol. (2005) 100:112–9. 10.1016/j.tripleo.2004.09.007 15953925

[41] Kristerson L. Autotransplantation of human premolars: a clinical and radiographic study of 100 teeth. Int J Oral Surg. (1985) 14:200–13. 10.1016/S0300-9785(85)80093-4 3920164

[42] Louropoulou A Andreasen JO Leunisse M Eggink E Linssen M Van der Weijden F An evaluation of 910 premolars transplanted in the anterior region—a retrospective analysis of survival, success, and complications. Dent Traumatol. (2024) 40:22–34. 10.1111/edt.12887 37731296

[43] Anitua E Mendinueva-Urkia M Galan-Bringas S Murias-Freijo A Alkhraisat MH. Tooth autotransplantation as a pillar for 3D regeneration of the alveolar process after severe traumatic injury: a case report. Dent Traumatol. (2017) 33:414–9. 10.1111/edt.12354 28627032

[44] Paulsen HU Andreasen JO Schwartz O. Pulp and periodontal healing, root development and root resorption subsequent to transplantation and orthodontic rotation: a long-term study of autotransplanted premolars. Am J Orthod Dentofacial Orthop. (1995) 108:630–40. 10.1016/S0889-5406(95)70009-9 7503041

[45] Zachrisson BU Stenvik A Haanæs HR. Management of missing maxillary anterior teeth with emphasis on autotransplantation. Am J Orthod Dentofacial Orthop. (2004) 126:284–8. 10.1016/j.ajodo.2004.06.007 15356486

[46] Badalyan VA Zedgenidze AM. Success factors for dental autotransplantation. Stomatologiya. (2020) 99:81. 10.17116/stomat20209904181 32692526

[47] de Freitas Coutinho NB Nunes FC Gagno Intra JB Roldi A de-Jesus-Soares A Coelho MS Success, survival rate, and soft tissue esthetic of tooth autotransplantation. J Endod. (2021) 47:391–6. 10.1016/j.joen.2020.11.013 33245974

[48] Cho JH Hwang HS Chang HS Hwang YC. Application of orthodontic forces prior to autotransplantation—case reports. Int Endod J. (2013) 46:187–94. 10.1111/j.1365-2591.2012.02116.x 22900919

[49] Akhlef Y Hosseini M Schwartz O Andreasen JO Gerds TA Jensen SS. Autotransplantation of premolars to the anterior maxilla: a long-term retrospective cohort study of survival, success, esthetic, and patient-reported outcome with up to 38-year follow-up. Dent Traumatol. (2025) 41:322–37. 10.1111/edt.13025 39663542

[50] Hou R Hui X Xu G Li Y Yang X Xu J Use of 3D printing models for donor tooth extraction in autotransplantation cases. BMC Oral Health. (2024) 24:179. 10.1186/s12903-024-03864-z 38311720 PMC10838442

[51] Dhillon IK Khor MMY Tan BL Wong RCW Duggal MS Soh SH Tooth autotransplantation with 3D-printed replicas as part of interdisciplinary management of children and adolescents: two case reports. Dent Traumatol. (2023) 39:81–9. 10.1111/edt.12837 36912701

[52] Wu Y Chen J Xie F Liu H Niu G Zhou L. Autotransplantation of mature impacted tooth to a fresh molar socket using a 3D replica and guided bone regeneration: two years retrospective case series. BMC Oral Health. (2019) 19:248. 10.1186/s12903-019-0945-8 31727038 PMC6857220

[53] Han S Wang H Chen J Zhao J Zhong H. Application effect of computer-aided design combined with three-dimensional printing technology in autologous tooth transplantation: a retrospective cohort study. BMC Oral Health. (2022) 22:5. 10.1186/s12903-021-02030-z 35012525 PMC8751250

[54] Companioni S Nguyen T Divaris K Christensen J. Optimizing donor tooth selection for autotransplantation in the anterior maxilla via CBCT-based root width and crown-root angle measurements. Dent Traumatol. (2023) 39:63–9. 10.1111/edt.12847 37060204

[55] Juslin J Teerijoki-Oksa T Jääsaari P Ekholm M Vallittu P Lassila L The accuracy of the CBCT-based 3-dimensional replica of the donor tooth in autotransplantation. Clin Exp Dent Res. (2024) 10:e70032. 10.1002/cre2.70032 39470682 PMC11520792

[56] Saiter Assis Beltrame L Delatorre Bronzato J Jacy da Silva Almeida T Batista Gagno Intra J Carneiro Nunes F de Freitas Britto C Evaluation of bone growth around autotransplanted teeth using cone-beam computed tomographic images. J Endod. (2024) 50:590–5. 10.1016/j.joen.2024.02.016 38428806

[57] Gilijamse M Baart JA Wolff J Sándor GK Forouzanfar T. Tooth autotransplantation in the anterior maxilla and mandible: retrospective results in young patients. Oral Surg Oral Med Oral Pathol Oral Radiol. (2016) 122:e187–92. 10.1016/j.oooo.2016.06.013 27492566

[58] Berlin-Broner Y Torrealba Y Flores-Mir C Levin L. Multidisciplinary approach for autotransplantation and restoration of a maxillary premolar into an area of an avulsed anterior tooth: a case report with a 6-year follow-up. J Endod. (2023) 49:590–6. 10.1016/j.joen.2023.02.007 36841382

[59] Plakwicz P Andreasen JO Górska R Burzykowski T Czochrowska E. Status of the alveolar bone after autotransplantation of developing premolars to the anterior maxilla assessed by CBCT measurements. Dent Traumatol. (2021) 37:691–8. 10.1111/edt.12680 33942473 PMC8453749

[60] Plakwicz P Czochrowska EM Milczarek A Zadurska M. Vertical bone growth following autotransplantation of the developing maxillary third molar to replace a retained mandibular permanent molar: a case report. Int J Periodontics Restorative Dent. (2014) 34:667–71. 10.11607/PRD.1984 25171037

[61] Thilander B Ödman J Gröteborg K Friberg B. Osseointegrated implants in adolescents. An alternative in replacing missing teeth? Eur J Orthod. (1994) 16:84–95. 10.1093/ejo/16.2.84 8005203

[62] Mishra S Chowdhary N Chowdhary R. Dental implants in growing children. J Indian Soc Pedod Prev Dent. (2013) 31:3–9. 10.4103/0970-4388.112392 23727735

[63] Ambrósio MFS Cançado RP Oliveira D Masioli BCG Cunha MA L D. Dental autotransplantation as a alternative treatment for the loss of permanent anterior teeth in children. Dental Press J Orthod. (2022) 27:e22spe4. 10.1590/2177-6709.27.4.E22SPE4 36169499 PMC9507021

[64] Gaviño Orduña JF García García M Dominguez P Caviedes Bucheli J Martin Biedma B Abella Sans F Successful pulp revascularization of an autotransplantated mature premolar with fragile fracture apicoectomy and plasma rich in growth factors: a 3-year follow-up. Int Endod J. (2020) 53:421–33. 10.1111/IEJ.13230 31587320

[65] Mena-Álvarez J Riad-Deglow E Quispe-López N Rico-Romano C Zubizarreta-Macho A. Technology at the service of surgery in a new technique of autotransplantation by guided surgery: a case report. BMC Oral Health. (2020) 20:99. 10.1186/S12903-020-01095-6 32264867 PMC7140495

[66] Singh AK Khanal N Acharya N Hasan MR Saito T Singh AK What are the complications, success and survival rates for autotransplanted teeth? An overview of systematic reviews and metanalyses. Healthcare. (2022) 10:835. 10.3390/HEALTHCARE10050835 35627972 PMC9141500

[67] Gostilo D Jākobsone G. Criteria and prognostic factors for survival and success rates for autotransplantation in the area of first and second molars: a systematic review and meta-analysis. Swiss Dent J. (2025) 135:145–74. 10.61872/sdj-2025-01-05 40072289

[68] Xia J Ge Z Zhang Y Shi J Xie Z. Prognostic factors for autotransplanted third molars with completely formed roots: a retrospective cohort study. J Am Dent Assoc. (2025) 156:46–56. 10.1016/j.adaj.2024.10.004 39580736

[69] Gonzalez-Ocasio J Stevens M. Autotransplantation of third molars with platelet-rich plasma for immediate replacement of extracted non-restorable teeth: a case series. J Oral Maxillofac Surg. (2017) 75:1833.e1–.e6. 10.1016/j.joms.2017.04.018 28529149

[70] Jakobsen C Stokbro K Kier-Swiatecka E Ingerslev J Thorn JJ. Autotransplantation of premolars: does surgeon experience matter? Int J Oral Maxillofac Surg. (2018) 47:1604–8. 10.1016/j.ijom.2018.08.003 30170776

[71] Mensink G Van Merkesteyn R. Autotransplantation of premolars. Br Dent J. (2010) 208:109–11. 10.1038/sj.bdj.2010.102 20147924

[72] Mendoza-Mendoza A Solano-Reina E Iglesias-Linares A Garcia-Godoy F Abalos C. Retrospective long-term evaluation of autotransplantation of premolars to the central incisor region. Int Endod J. (2012) 45:88–97. 10.1111/j.1365-2591.2011.01951.x 21906087

[73] Yoshino K Kariya N Namura D Noji I Mitsuhashi K Kimura H Survival rate in autotransplanted premolars with complete root formation: a retrospective clinical survey clinical report. Bull Tokyo Dent Coll. (2013) 54(1):27–35. 10.2209/tdcpublication.54.27 23614950

[74] Kamata Y Shimizu T Tsunoda A Tamura T Komaki M Kodama T. Periodontal tissue healing after autologous tooth transplantation: a retrospective analysis of case series. Oral Health Prev Dent. (2021) 19:619–26. 10.3290/j.ohpd.b2403411 34874139 PMC11640961

[75] Marques-Ferreira M Rabaça-Botelho MF Carvalho L Oliveiros B Palmeirão-Carrilho EV. Autogenous tooth transplantation: evaluation of pulp tissue regeneration. Med Oral Patol Oral Cir Bucal. (2011) 16:e984–9. 10.4317/medoral.16926 21743431

[76] Elagib MFA Alqaysi MAH Almushayt MOS Nagate RR Gokhale ST Chaturvedi S. Dental implants in growing patients: a systematic review and meta-analysis. Technol Health Care. (2023) 31:1051–64. 10.3233/THC-220581 36502352

[77] García-Miralles E Marqués-Martínez L Borrell-García C Boo-Gordillo P Aura-Tormos JI Guinot-Barona C. Tooth autotransplantation with immature donors in children and adolescents: a systematic review with quality-assessed evidence. J Clin Med. (2025) 14:8387. 10.3390/JCM14238387/S1 41375692 PMC12693242

[78] Meto A Çota K Meto A Bara S Boschini L. Tooth autotransplantation in contemporary dentistry: a narrative review of its clinical applications and biological basis. J Clin Med. (2025) 14:6249. 10.3390/JCM14176249 40944008 PMC12429413

[79] Mankani N Chowdhary R Patil BA Nagaraj E Madalli P. Osseointegrated dental implants in growing children: a literature review. J Oral Implantol. (2014) 40:627–31. 10.1563/AAID-JOI-D-11-00186 22214484

[80] Yang Y Bai Y Li S Li J Gao W Ru N. Effect of early orthodontic force on periodontal healing after autotransplantation of permanent incisors in beagle dogs. J Periodontol. (2012) 83:235–41. 10.1902/jop.2011.110099 21692629

